# Comparing nutrient intake and body weight status amongst adolescent substance users, institutionalised abstainers and never users

**DOI:** 10.29219/fnr.v63.3634

**Published:** 2019-12-19

**Authors:** Tony Ka-chun Yung, Joseph Tak-fai Lau

**Affiliations:** Jockey Club School of Public Health and Primary Care, Faculty of Medicine, The Chinese University of Hong Kong, Hong Kong SAR, China

**Keywords:** young people, substance use, dietary intake, weight reduction, ketamine

## Abstract

**Background:**

Improved nutrition intake in drug rehabilitation programmes enhances quality sobriety and prevents relapses. However, little is known about the nutritional status of substance users and institutionalised abstainers. Previous nutritional studies have mainly focused on methamphetamine, whereas ketamine has not been investigated despite its popularity amongst adolescents.

**Objectives:**

To compare nutrient intake and underweight status amongst three groups of adolescents –current substance users, institutionalised abstainers and never users (controls) – and examine the association between ketamine use and nutrient intake.

**Design:**

This is a cross-sectional questionnaire survey which was conducted using face-to-face interview. Substance users (*n* = 202) and never users (*n* = 100) were invited through the outreach social workers of three non-government organisations. Abstainers (*n* = 50) were recruited from three drug rehabilitation centres. Nutrient intake was assessed through two 24-h recalls. Other information collected included anthropometrics, socio-demographic characteristics and substance type used over the previous month.

**Results:**

Only 20.8 and 15.9% of male and female substance users met the daily energy requirements. Male users were less likely to meet the recommended intake of energy [odds ratio (OR) = 0.37] and protein (OR = 0.10) than controls. Overall, abstainers had better intake of beneficial nutrients than substance users. However, abstainers were more likely to overconsume harmful nutrients, such as cholesterol and sodium. Regarding weight status, female substance users (56.1%) were more likely to be underweight than abstainers (14.8%) (OR = 8.85). Amongst underweight female substance users, 52.2% were still trying to lose more weight. Moreover, ketamine users tended to have lower intake of nutrients from animal sources than the users of other drugs.

**Conclusions:**

Adolescent substance users are at risk of energy and nutrient inadequacy. Misconceptions about body weight are disseminating amongst them. The study findings provide valuable information for frontline workers taking care of young substance users and for institutions providing residential rehabilitation programmes.

## Popular scientific summary

- Adolescent substance users are at risk of energy and protein inadequacy.
- More than half of female substance users are underweight, of which more than half are still trying to lose weight.
- Users of ketamine tend to have lower intake of nutrients from animal sources than users of other drugs.

Drug abuse adversely affects nutritional status (1, 2). Substance use may change brain function, thereby causing mood disorders and worsening appetite (3). It also affects oral health, such as reduced chewing strength, resulting in indigestion (4). A previous study showed that over two-thirds of sampled drug addicts exhibit different levels of anorexic symptoms, and that the mean caloric intakes of male and female addicts are below 50% of the recommended daily intake level (5). The low dietary intakes of vitamin B6, vitamin B12, selenium and zinc are also found amongst severe drug abusers (6). Amongst hospitalised drug-poisoned patients, 76.1% present unsatisfactory nutritional status (7). Nutritional deficiency along with drug abuse increases the risk of developing metabolic syndrome due to the reduced energy and antioxidant potential of body cells (2). This chronic metabolic disorder is a risk factor of chronic diseases, such as diabetes and cardiovascular diseases (2). The harmful effect of drug abuse is of particular concern to adolescents given that poor dietary intake and nutritional status cause irreversible harm to their cognitive and physical development (8). Nevertheless, existing literatures concerning this population only focus on the association between drug use and food item intake (9, 10). No study has investigated the dietary intake of adolescent substance users at the nutrient level.

In view of the negative health impact associated with long-term nutrition impairment, the American Dietetic Association calls for the improvement of food and nutrition intake in drug rehabilitation programmes, claiming that this action would enhance the quality of sobriety and prevent relapse (11). Substance treatment and rehabilitation programmes, especially programmes offering residential services, are warranted to provide quality nutrition education and food nourishment to participants. Information on the nutritional intake and body weight status of adolescent drug addicts is essential as the improvement of these two factors can help prevent relapse. An understanding of the existing status of the above-mentioned two factors is needed so that residential rehabilitation programmes can improve their food service.

Previous nutritional studies have mainly focused on dietary intakes amongst methamphetamine abusers (2, 12, 13). Other types of psychotropic substances have been poorly investigated. Amongst these psychotropic substances, ketamine is the most used substance in Hong Kong and in other countries and is popular amongst adolescents (14, 15). Therefore, we focused on the association of ketamine with dietary intake

This study aims to investigate and compare the dietary intake, including energy, beneficial nutrients and potentially harmful nutrients, and body weight status amongst three groups of adolescents in Hong Kong, namely, current substance users, institutionalised abstainers and a control group of never users. We also investigated the nutrient intake of ketamine users and compared it with that of the users of other psychotropic drugs.

## Methods

### Participants and data collection

Three groups of adolescents participated in this study: (1) current substance users who self-reported having used psychotropic drugs at least once a week in the previous month; (2) institutionalised abstainers and (3) a control group who self-reported having never used psychotropic drugs. The psychotropic drugs considered in this study included ketamine, methylamphetamine, cocaine, cannabis, ecstasy, nimetazepam, flunitrazepam, triazolam, midazolam, zopiclone, Lysergic acid diethylamide (LSD) and codeine. Chinese adolescents aged 18 years or younger at the time of the first interview were eligible for the study.

Adolescent substance users were recruited through outreach social workers of three collaborating non-government organisations (NGOs) serving youths with behavioural problems. They were invited during routine outreach service. The recruited participants met twice with the study’s fieldworker at the activity centres of the participating NGOs. The fieldworker was experienced in social research and trained by the study investigator, who was blinded for review, on dietary data collection. During the first meeting, a briefing was made, and written informed consent was obtained from the study participants. A face-to-face interview, which included dietary assessment, was then conducted at a private location. The body weight and height of the participants were measured on-site. The second meeting occurred 1 week after to complete the second dietary assessment (see the ‘Measures’ section) and to clarify any unclear responses obtained during the first interview.

Amongst the 219 eligible adolescent substance users referred to the research team by social workers, 17 completed only the first interview. The remaining 202 substance users completed both interviews, and their data were analysed. Upon the completion of the second interview, each participant was given a supermarket coupon with HK$150 cash value as a token of appreciation for their time. All interviews were anonymous. Participants were assured that participation in the study was totally voluntary, and the data obtained would be used for research purposes only and would not be disclosed to any other parties, including the social worker caring for them. Research ethics approval was obtained from the Survey and Behavioural Research Ethics Committee of the Chinese University of Hong Kong. This study was supported by the Beat Drugs Fund (Project no. BDF090067) of the Narcotics Division of Hong Kong SAR Government.

The same strategy as mentioned above was used to recruit the members of the control group (never users). Recruitment was followed by the same consent and interview procedures for data collection. A total of 100 controls completed the first and second interviews. They received a cash coupon of HK$20 as a token of appreciation for participating in the study.

The participants of the abstainer group were recruited from three institutional drug rehabilitation centres (two male centres and one female centre). The research team sent invitations to all potential centres in the territory, and only three centres responded positively. A total of 60 and 30 abstainers were staying in the two male centres, and 20 abstainers were staying in the female centre. The participants would usually stay for 6–9 months at these centres. They are provided with three main meals free of charge with fruits. Snacks between meals are available at their own expense. Fifty abstainers were invited by the centre staff to participate in the study on a voluntary basis. All of the invited abstainers consented to participate and completed the two aforementioned face-to-face interviews. The participants were informed that participation is voluntary and that refusal to participate in the study would not affect the services that they were going to receive at the centre. Similar briefing and data collection procedures were provided, with the exception that no incentives were given to this group.

## Measures

Participants were asked about the types of substances they used during the last month. Sociodemographic characteristics, including age, employment status and family income, were asked. Participants were also asked about their self-perceived health and nutritional and weight status and whether they were trying to increase or lose their body weight. Dietary intakes were assessed through two 24-h dietary recalls. To account for day-to-day variation in dietary intake, the first recall was based on a weekday, and the second recall was based on a weekend. Previous studies have shown that 24-h recall has advantages over the food frequency questionnaire and a 3-day food diary in assessing dietary intake amongst drug users (16). Body weight was measured using a portable electronic balance, and height was measured by a stadiometer using standardised procedures.

## Nutritional analyses

Data from the two 24-h dietary recalls were analysed using the Food Processor Nutrition Analysis V8.0 software (ESHA Research, Salem, OR, USA). Additional recipes were added to the original database to analyse local food items. The average nutrient intakes obtained from the two recalls were used for data analysis. The recommended energy intake level was derived from the age- and gender-fit Schofield equation adjusted for the participants’ activity levels (17). In addition, five beneficial nutrients (dietary fibre, protein, iron, calcium and vitamin C) and four potentially harmful nutrients (total fat, saturated fat, cholesterol and sodium) were considered in the assessment of the participants’ dietary intake. The selection of these nutrients was based on the Diet Quality Index – International (18) and the Hong Kong Population-Based Food Consumption Survey conducted from 2005 to 2007 (19). The recommended levels of these nutrients were derived from age- and gender-specific US Dietary Reference Intakes (DRI) values (20). The intake levels of macronutrients, such as protein, total fat and saturated fat, were energy-adjusted. For beneficial nutrients, the aim was to meet the minimum DRI requirement. For potentially harmful nutrients, the criterion used was that they do not exceed the recommended limits.

## Statistical analyses

Chi-squared tests were used to compare the differences in demographics, energy and nutrient intakes between male and female participants. Univariate and multivariate logistic regression models were fitted to investigate the associations between the status of substance use and the following: (1) whether participants met the recommended beneficial nutrients, (2) whether participants exceeded the recommended limit of potential harmful nutrients, (3) underweight status and (4) self-perceived underweight status. Multivariate logistic regression models were adjusted for age, employment status and family income. The same model was fitted to assess the associations between ketamine use and the above-mentioned dependent variables. Statistical significance was considered at *P* < 0.05. All analyses were performed using SPSS version 16.0 (SPSS Inc., Illinois, USA).

## Results

### Characteristics and dietary intake of the study sample

A total of 352 participants completed the two interviews (substance users: *n* = 202; never users: *n* = 100; and institutionalised abstainers: *n* = 50). Ketamine was used by 75.7% of substance users. More male substance users (80.8%) used ketamine over the previous month compared to female substance users (68.3%) (*P* = 0.04; Table 1). In the entire study sample, 91.7% of participants were between 13 and 18 years old, 63.1% were students and 36.4% were underweight. More females (46.6%) than males (23.9%) perceived themselves to be overweight (*P* <0.001).

**Table 1:** Characteristics and dietary intake of study sample (*n* = 352)

Factors	Male (%) (*n* = 197)	Female (%) (*n* = 155)	*P* for χ2	Overall (%) (*n* = 352)
**Type of respondents**				
Drug use status				
User	60.9	52.9	0.21	57.4
Control	27.4	29.7		28.4
Abstainer	11.7	17.4		14.2
Type of drug use (amongst drugs users)				
Ketamine users	80.8	68.3	**0.04**	75.7
Non-ketamine users	19.2	31.7		24.3
**Socio-demographics**				
Age (years)				
9–12	7.6	9	0.53	8.2
13–15	38.6	43.2		40.6
16–18	53.8	47.7		51.1
Employment status				
Student	62.9	63.2	**0.02**	63.1
Unemployed	19.8	28.4		23.6
Working	17.3	8.4		13.4
Monthly family income ($)				
<10,000	12.2	11.6	**0.01**	11.9
10,001–30,000	34.8	20.4		28.4
>30,000	3.9	0		2.1
Receiving government subside	4.4	8.2		6.1
Don’t know	44.8	59.9		51.5
**Body weight status**				
Underweight	31.5	42.6	**0.01**	36.4
Normal	51.8	51		51.4
Overweight	16.8	6.5		12.2
**Self-perceived body weight status**				
Underweight	22.2	14.2	**< 0.001**	18.6
Normal	53.9	39.2		47.3
Overweight	23.9	46.6		34.1
**Dietary intake**				
** Energy (% meeting the reference intake)**	26.4	21.9	0.33	24.4
**Beneficial nutrients (% meeting the reference intake)**				
Dietary fibre	7.1	16.8	**0.01**	11.4
Protein	61.9	42.6	**< 0.001**	53.4
Iron	24.9	10.3	**< 0.001**	18.5
Calcium	19.8	15.5	0.3	17.9
Vitamin C	13.7	24.5	**0.01**	18.5
**Harmful nutrients (% not exceeding the reference intake)**				
Total fat	58.4	61.9	0.5	59.9
Saturated fat	71.1	76.1	0.29	73.3
Cholesterol	65	81.3	**0.01**	72.2
Sodium	37.6	57.4	**< 0.001**	46.3

In terms of dietary intake, only 26.4% males and 21.9% females met the recommended energy intake requirement. The percentage of participants fulfilling the recommended intake level of beneficial nutrients ranged from 7.1% (dietary fibre) to 61.9% (protein) for males and from 10.3% (iron) to 42.6% (protein) for females. The prevalence of participants keeping their intakes of potentially harmful nutrients below the recommended limit ranged from 37.6% (sodium) to 71.1% (saturated fat) for males and from 57.4% (sodium) to 81.3% (cholesterol) for females. Male and female respondents were significantly different in meeting the reference intake level of dietary fibre, protein and iron and not exceeding the reference intake of cholesterol and sodium (all *P* < 0.05; Table 1).

### Comparison of dietary intakes and body weight status amongst the three groups of participants

#### Substance users versus control

Male substance users were less likely than never users to meet the recommended intake level of energy [20.8% vs. 42.6%; adjusted odds ratio – AOR (95% confidence interval – CI) = 0.37 (0.16–0.87)] and protein [46.7% vs. 85.2%; AOR (95% CI) = 0.10 (0.04–0.28)] (Table 2). Male [30.0% vs. 35.2 %, AOR (95% CI) = 1.01 (0.45–2.28)] and female [56.1% vs. 34.8%, AOR (95% CI) = 2.06 (0.92–4.62)] substance users showed insignificant differences in body weight as compared with controls (Table 3). Amongst underweight substance users, only 50% of males and 32.6% of females rightly perceived themselves to be underweight, and 5.6% of males and 52.2% of females were still trying to lose weight (Tables 2 and 3).

**Table 2:** Association between substance use status and nutrient intake/body weight factors amongst male respondents (*n* = 197)

				Adjusted OR (95% CI)*		
	User (*n* = 120)	Abstainer (*n* = 23)	Control (*n* = 54)	User vs. Control^#^	User vs. Abstainer^#^	Abstainer vs. Control^#^
**Energy (% meeting the reference intake)**	20.8	17.4	42.6	**0.37 (0.16–0.87)**	1.00 (0.30–3.37)	**0.13(0.02–0.73)**
**Beneficial nutrient (% meeting the reference intake)**						
Dietary fiber	5	4.3	13	0.68 (0.17–2.68)	1.03 (0.11–9.59)	0.15 (0.01–1.64)
Protein	46.7	87	85.2	**0.10 (0.04–0.28)**	**0.12 (0.03**–**0.45)**	0.24 (0.04–1.53)
Iron	27.5	8.7	25.9	0.96 (0.40–2.32)	3.21 (0.69–14.96)	0.21 (0.03–1.29)
Calcium	13.3	60.9	16.7	0.65 (0.23–1.89)	**0.10 (0.04**–**0.29)**	**9.90 (2.43**–**40.30)**
Vitamin C	15.8	13	9.3	1.69 (0.51–5.58)	1.09 (0.28–4.19)	1.28 (0.23–7.16)
**Harmful nutrients (% not exceeding the reference intake)**						
Total fat	64.2	56.5	46.3	1.55 (0.72–3.33)	1.48 (0.58–3.77)	2.50 (0.69–9.12)
Saturated fat	75.8	65.2	63	1.60 (0.70–3.65)	1.72 (0.64–4.64)	1.53 (0.40–5.85)
Cholesterol	65.8	60.9	64.8	1.08 (0.49–2.41)	1.33 (0.51–3.46)	3.29 (0.66–16.46)
Sodium	41.7	8.7	40.7	1.24 (0.57–2.68)	**7.16 (1.58**–**32.47)**	0.30 (0.06–1.61)
**Body weight factor (%)**						
Being underweight	30	30.4	35.2	1.01 (0.45–2.28)	0.91 (0.33–2.51)	1.86 (0.50–6.85)
Self-perceived underweight (amongst those who are underweight)	50	100	42.1	0.74 (0.17–3.12)	N/A	N/A
Trying to lose weight (amongst those who are underweight)	5.6	0	0	N/A	N/A	N/A

# Reference group;

* Adjusted for age, employment status and family income.

**Table 3:** Association between substance use status and nutrient intake/body weight factors amongst female respondents (*n* = 155)

				Adjusted OR (95% CI)*		
	User (*n* = 82)	Abstainer (*n* = 27)	Control (*n* = 46)	User vs. Control^#^	User vs. Abstainer^#^	Abstainer vs. Control^#^
**Energy (% meeting the reference intake)**	15.9	33.3	26.1	0.65 (0.25–1.70)	**0.28 (0.09**–**0.83)**	2.75 (0.67–11.30)
**Beneficial nutrient (% meeting the reference intake)**						
Dietary fibre	11	51.9	6.5	2.17 (0.51–9.21)	**0.07 (0.02**–**0.24)**	**11.86 (2.34**–**22.32)**
Protein	29.3	77.8	45.7	0.73 (0.32–1.68)	**0.07 (0.02**–**0.24)**	**31.24 (2.88**–**338.40)**
Iron	6.1	18.5	13	0.70 (0.17–2.83)	**0.22 (0.05**–**0.95)**	2.84 (0.49–16.38)
Calcium	8.5	44.4	10.9	0.63 (0.16–2.42)	**0.09 (0.03**–**0.30)**	**22.42 (3.35**–**150.06)**
Vitamin C	18.3	55.6	17.4	1.32 (0.47–3.69)	**0.07 (0.02**–**0.26)**	**86.53 (5.14**–**1455.98)**
**Harmful nutrients (% not exceeding the reference intake)**						
Total fat	63.4	66.7	56.5	1.79 (0.78–4.13)	0.74 (0.28–1.94)	2.12 (0.56–8.04)
Saturated fat	76.8	81.5	71.7	1.86 (0.72–4.77)	0.59 (0.19–1.90)	2.15 (0.48–9.63)
Cholesterol	85.4	74.1	78.3	1.06 (0.38–2.95)	**3.81 (1.10**–**13.14)**	**0.13 (0.02**–**0.71)**
Sodium	64.6	33.3	58.7	1.01 (0.45–2.30)	**5.22 (1.89**–**14.38)**	**0.15 (0.03**–**0.69)**
**Body weight factor (%)**						
Being underweight	56.1	14.8	34.8	2.06 (0.92–4.62)	**8.85 (2.67**–**29.31)**	0.33 (0.07–1.48)
Self-perceived underweight (amongst those who are underweight)	32.6	33.3	12.5	2.47 (0.44–13.80)	1.53 (0.10–23.12)	2.33 (0.56–10.32)
Trying to lose weight (amongst those who are underweight)	52.2	25	37.5	2.20 (0.59–8.20)	4.00 (0.35–46.25)	0.18 (0.01–5.13)

# Reference group;

* Adjusted for age, employment status and family income.

#### Substance users versus abstainers

Generally speaking, current substance users were less likely to meet the recommended intake of beneficial nutrients than institutionalised abstainers. Male substance users were less likely to meet the recommended intake for protein [46.7% vs. 87.0%, AOR (95% CI) = 0.12 (0.03–0.45)] and calcium [13.3% vs. 60.9%, AOR (95% CI) = 0.10 (0.04–0.29)] than abstainers (Table 2). Female substance users were less likely than abstainers to meet the recommended intake of energy [15.9% vs. 33.3%, AOR (95% CI) = 0.28 (0.09–0.83)], dietary fibre [11.0% vs. 51.9%, AOR (95% CI) = 0.07 (0.02–0.24)], protein [29.3% vs. 77.8%, AOR (95% CI) = 0.07 (0.02–0.24)], iron [6.1% vs. 18.5%, AOR (95% CI) = 0.22 (0.05–0.95)], calcium [8.5% vs. 44.4%, AOR (95% CI) = 0.09 (0.03–0.30)] and vitamin C [18.3% vs. 55.6%, AOR (95% CI) = 0.07 (0.02–0.26)] (Table 3). Compared with abstainers, current substance users were more likely to maintain the intake levels of potentially harmful nutrients within the recommended limits [male substance users vs. abstainers: sodium [41.7% vs. 8.7%, AOR (95% CI) = 7.16 (1.58–32.47)]; female substance users vs. abstainers: cholesterol [85.4% vs. 74.1%, AOR (95% CI) = 3.81 (1.10–13.13)] and sodium [64.6% vs. 33.3%, AOR (95% CI) = 5.22 (1.89–14.38)]. Male substance users and abstainers had similar prevalence of being underweight [30.0% vs. 30.4%, AOR (95% CI) = 1.01 (0.45–2.28)]. Female substance users were more likely to be underweight than abstainers [56.1% vs. 14.8%, AOR (95% CI) = 8.85 (2.67–29.31)] (Tables 2 and 3).

#### Abstainers versus control

Male abstainers were less likely than never users to meet energy requirements [AOR (95% CI) = 0.13 (0.02–0.73)]. However, they were more likely to meet calcium requirements [AOR (95% CI) = 9.90 (2.43–40.30)] than never users. For females, with the exception of iron, abstainers were more likely to meet the recommended intake for the beneficial nutrients of dietary fibre, protein, calcium and vitamin C (AOR from 11.86 to 22.42) than users. However, they were less likely than users to maintain the recommended intake of cholesterol and sodium (AOR from 0.13 to 0.15) (Tables 2 and 3).

### Associations between ketamine use and dietary intake amongst substance users

Compared with users of other substances, ketamine users were less likely to meet the intake requirement for protein [37.3% vs. 46.9%, AOR (95% CI) = 0.49 (0.24–0.98)] and iron [16.3% vs. 26.5%, AOR (95% CI) = 0.35 (0.15–0.82)] but were more likely to maintain cholesterol intake within the recommended limit [75.8% vs. 67.3%, AOR (95% CI) = 2.18 (1.01–4.70)]. (Table 4)

**Table 4:** Associations between ketamine use and nutrient intake amongst substance users (*n* = 202)

	Non-ketamine user^#^ (*n* = 49)	Ketamine user (*n* = 153)	Adjusted OR (95% CI)*
**Energy (% meeting the reference intake)**	22.4	17.6	0.59 (0.26–1.35)
**Beneficial nutrient (% meeting the reference intake)**			
Dietary fibre			
Protein	10.2	6.5	0.69 (0.22–2.23)
Iron	46.9	37.3	**0.49 (0.24**–**0.98)**
Calcium	26.5	16.3	**0.35 (0.15**–**0.82)**
Vitamin C	14.3	10.5	0.68 (0.25–1.82)
	20.4	15.7	0.78 (0.34–1.81)
**Harmful nutrients (% not exceeding the reference intake)**			
Total fat	73.5	60.8	0.56 (0.27–1.16)
Saturated fat	85.7	73.2	0.49 (0.20–1.19)
Cholesterol	67.3	75.8	**2.18 (1.01**–**4.70)**
Sodium	59.2	48.4	0.77 (0.39–1.53)

# Reference group for OR;

* Adjusted for gender, age, employment status and family income.

## Discussion

One major finding is that adolescent substance users had inadequate intakes of energy, protein and some beneficial nutrients. Only 20.8 and 15.9% of male and female substance users met the daily energy intake requirement, respectively. Male users were significantly less likely to meet energy and protein requirements than the control group. This result contradicts the findings of similar studies that focused on young substance users. Arcan et al. (10) revealed that substance abuse is positively associated with high-fat food intake amongst adolescents attending alternative high schools. Nolan (9) showed that the frequency and breadth of substance use strongly predict elevated food consumption and ratings of hunger and desire to eat amongst college students. The present study did not recruit the users of a specific drug type. Thus, making direct comparisons with the above-mentioned studies is impossible. However, the results of the current study provided evidence that drug abuse could potentially lead to calorie and protein deficiency amongst adolescents. The low levels of energy and protein intakes may or may not represent the direct effect of drug use. However, the observation is noteworthy because such inadequacy may aggregate the harmful effects of substance use (2). Adolescents are undergoing an accelerated phase of physical, intellectual and emotional development; thus, they require an adequate supply of energy and nutrients (21). Protein is essential for the formation of growth hormones and antibodies, which are crucial for the growth of adolescents. Long-term inadequacy in the levels of energy and protein intake will retard adolescents’ growth and increase the risk of developing chronic diseases later in adulthood (22, 23).

The present study reveals that the problem of dietary inadequacy amongst adolescent substance users extends to micronutrients. The very low percentages of male drug users meeting the recommended intake levels of calcium and the very low percentages of female drug users meeting the recommended intake levels of iron and calcium are of particular concern. Calcium is the building block of growing bones. Low calcium intake increases the risk of osteoporosis later in life. Moreover, long-term calcium deficiency increases the risk of developing colorectal cancer (24). Only 6.1% of female substance users met the dietary requirement of iron intake. Although iron is crucial for blood cell formation and thus the maintenance of the menstrual cycle, iron deficiency anaemia is extremely common amongst adolescent girls in Hong Kong and other affluent societies (25, 26). Iron deficiency potentially increases the risk of infection and adverse pregnancy outcomes (27). Our findings on the low level of dietary iron intake amongst female respondents are consistent with those reported elsewhere (28, 29). Considering the results of the current study, health education campaigns are necessary to target adolescent substance users and relevant social workers to improve nutritional health literacy and promote healthy nutrition. Substance users at high risk or with symptoms of malnutrition should be referred to dieticians for individual counselling. Personalised counselling can provide good insight into an individual’s nutritional inadequacy and need, and it can also provide direct dietary regime and thus is helpful in reducing the risk of nutrient deficiencies.

Institutionalised abstainers were more likely to attain energy and beneficial nutrient recommendations than current substance users. Institutionalisation ensures satisfactory food supply and improved dietary habits. The result of this study suggests that the problem of dietary inadequacy amongst adolescent substance users is modifiable and could be improved with proper arrangements. However, the excessive consumption of potentially harmful nutrients is a concern amongst abstainers. For instance, the proportion of female abstainers maintaining their cholesterol and sodium intake within the recommended limits was less than that of female substance users. A similar phenomenon was found when female abstainers were compared with controls, thereby suggesting that the nutritional value of the food supply can still improve. Moreover, the difference in results amongst male and female abstainers as compared with their counterparts may suggest that the quality of food supplied by different institutes may have large discrepancies. Further investigation on the nutrient content of foods supplied by these institutions is needed. Nutritionists or dieticians should provide nutrition education to adolescent substance abstainers and institutional staff and help design healthy menus for these institutions to ensure a satisfactory supply of beneficial nutrients and reduce potential harmful nutrients.

Echoing the observation of a poor dietary intake amongst substance users in the current study, a high percentage of substance users were underweight (30% males and 56.1% females). Many of the underweight substance users did not realise their weight problem. Even worse, more than half of the underweight female substance users were still trying to lose weight. Some misconceptions about body weight are being propagated amongst the population. A previous study revealed that some adolescent girls may use substances as a weight control strategy (30). This finding has important implications for antidrug campaigns and rehabilitation programmes and highlights the importance of rectifying the misconception about the relationship between psychotropic drugs and body weight. Future longitudinal studies are warranted to elucidate the relationship amongst dietary intake, body weight and perceived body image in young drug addicts. The results of these studies provide information required to develop nutrition intervention methods for this population.

Our results suggested that ketamine users were less likely to meet the recommended intake of protein and iron but were more likely to maintain the intake of cholesterol within the recommended limit than the users of other types of drugs. This is the first study to investigate such associations. Ketamine users in our study tended to consume less animal food compared with the users of other drugs. Animal studies have demonstrated the effect of ketamine on taste aversion (31, 32). However, human studies in this area remain scarce, although reduced appetite and nausea/vomiting are reported side effects of ketamine use. Given that ketamine is preferred by young substance users (14, 15), future studies are warranted to investigate the physiological effects of ketamine, particularly its effects on potential taste changes and food selection.

This study has several limitations. Firstly, the hidden nature of substance users made random sampling unfeasible. The convenience sampling method may have introduced selection bias. Secondly, the cross-sectional design prohibited the assessment of the causal relationship between study variables. For example, we could not confirm whether being underweight was a result of substance use or the other way round. Similarly, the association between ketamine use and dietary inadequacy cannot confirm which one represents the cause or consequence. Thirdly, body composition and biochemical indicators that provide a comprehensive picture of nutritional status were not measured. Finally, only three rehabilitation centres were involved, and the sample size of abstainers was small.
